# Particle Track and Trace during Membrane Filtration by Direct Observation with a High Speed Camera

**DOI:** 10.3390/membranes10040068

**Published:** 2020-04-10

**Authors:** Mads Koustrup Jørgensen, Kristian Boe Eriksen, Morten Lykkegaard Christensen

**Affiliations:** Center for Membrane Technology, Aalborg University, Fredrik Bajers Vej 7H, 9220 Aalborg Øst, Denmark; boe.eriksen.kbe@gmail.com (K.B.E.); mlc@bio.aau.dk (M.L.C.)

**Keywords:** microfiltration, fouling, monitoring, model particles, microscopy, image analysis

## Abstract

A methodology was developed for direct observation and analysis of particle movements near a microfiltration membrane. A high speed camera (1196 frames per second) was mounted on a microscope to record a hollow fiber membrane in a filtration cell with a transparent wall. Filtrations were conducted at varying pressure and crossflow velocities using synthetic core–shell particles (diameter 1.6 μm) of no and high negative surface charge. MATLAB scripts were developed to track the particle positions and calculate velocities of particle movements across and towards the membrane surface. Data showed that the velocity of particles along the membrane increases with distance from the membrane surface which correlates well with a fluid velocity profile obtained from CFD modelling. Particle track and trace was used to calculate the particle count profiles towards the membrane and document a higher concentration of particles near the membrane surface than in the bulk. Calculation of particle velocity towards and away from the membrane showed a region within 3–80 μm from the membrane surface with particle velocities higher than expected from the velocity of water through the membrane, thus the permeation drag underpredicts the actual velocity of particles towards the membrane. Near the membrane, particle velocities shift direction and move away. This is not described in classical filtration theory, but it has been speculated that this is an effect of particle rotation or due to membrane vibration or change in flow pattern close to the membrane.

## 1. Introduction

Crossflow microfiltration is an established unit operation in several applications from water and wastewater treatment to food and pharmaceutical industry. However, membrane performance is limited by membrane fouling. For microfiltration, the drag of particles and colloids with the permeate flow results in membrane pore blockage and cake layer formation, reducing the permeability of the membrane. Hence, higher transmembrane pressure (TMP) and frequent cleaning (physical or chemical) is required to maintain flow through the membrane [1]. Therefore, the mechanisms of cake formation and behavior has been studied in several studies in lab and pilot scale to understand fouling mechanisms and through that alleviate fouling. From that, mathematical models have been developed based on extended DLVO theory describing particle–particle and particle–membrane interactions [2], theories for cake filtration [3], and cake formation has been described with particle transport theories. The latter has been described for crossflow microfiltration by Ripperger and Altmann [4], dividing the forces acting on a particle or colloid:

- *F_G_* is the gravitational force of particles transported towards the membrane by permeation,
- *F_D_*, a drag force of particles being dragged across the membrane by the crossflow,
- *F_F_*, a frictional force of particles moving along the membrane acting against the crossflow, hence slowing down the particles as the move across the membrane, and
- *F_L_*, lifting force, acting opposite to the permeate flow as pressure increase when the water velocity decrease near the surface.

The mentioned models have been described theoretical and compared to filtration data in terms of permeate flux, TMP and “post mortem” analysis of membranes after filtration, e.g., SEM [5,6]. However, the development of on-line fouling monitoring techniques have added extra dimensions into understanding the mechanisms of fouling layer structure and formation [7]. Ultrasonic and laser based methods have been developed for indirect measurements of fouling layer thickness during filtration [8,9,10], while a more recent method, fluid dynamic gauging, can also estimate the cohesive strength of fouling layers [11,12]. Direct observation (DO) techniques have been developed to directly monitor the formation of fouling with videos recording the membrane through a microscope and follow the evolution of thickness [13,14,15,16] and to observe single layer formation [17]. However, the existing method is limited to observe deposited particles and thus cannot observe how the particles approach to the membrane. With high speed cameras it will be possible to observe the particles as they approach the membrane also at high cross-flow velocities. This enables the observation of particle distribution (concentration polarization), particle speed with μm precision and particle acceleration. This can contribute to a new understanding of the forces acting on particles before the membrane fouls, and thereby be used to study how crossflow velocity, TMP, particle and membrane characteristics affect particle transport and risk of fouling formation.

In this study, a high crossflow microfiltration cell is designed and connected with a high speed camera and a microscope to study and understand the transport of particles along and towards the membrane prior to membrane fouling. A MATLAB script will be developed to track particles and calculate concentration and velocity profiles along the membrane. Filtrations will be carried out at varying TMP, crossflow velocity and particle surface charges to understand their influence on particle behavior near the membrane. For this, synthetic microparticles with a hard core and a water swollen shell, developed in a previous study, are selected [17]. The particles are selected as they are spherical and monomodal in size distribution (mean diameter of 1.6 μm) and variable surface charge, making them ideal for controlled studies of microfiltration foulants behavior.

## 2. Materials and Methods 

### 2.1. Direct Observation Setup

**Figure 1:**
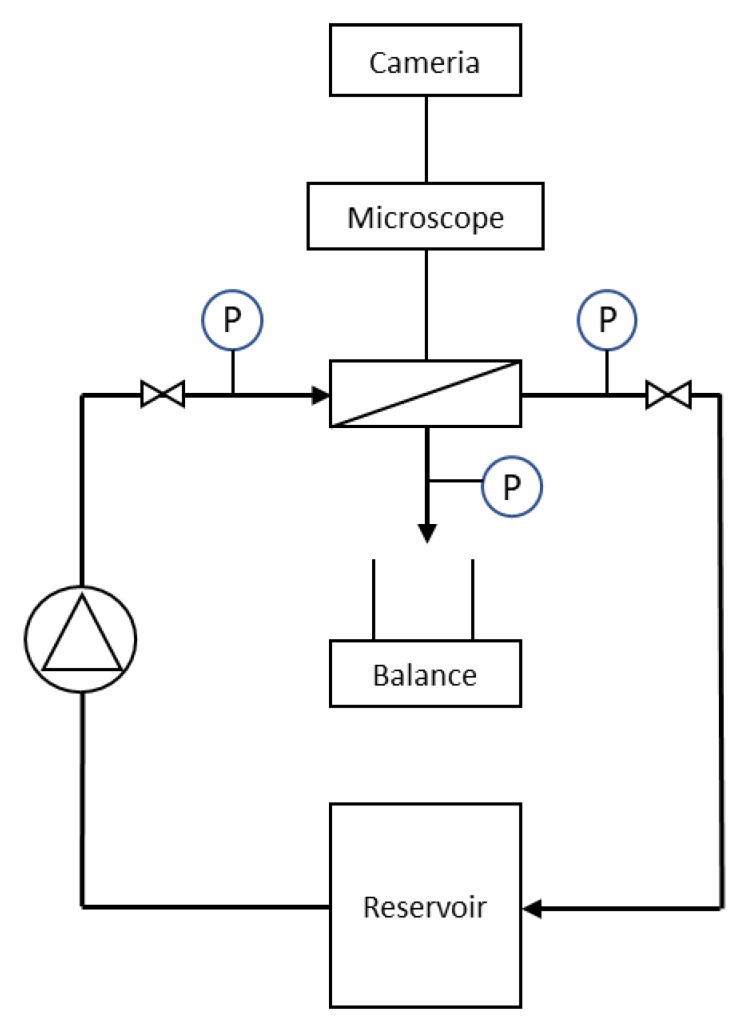
Schematic drawing of filtration system with camera and microscope connected to filtration cell and balance for collection of permeate.

A DO apparatus was developed. The DO apparatus consisted of a custom crossflow membrane module, a microscope (10× objective lens, Carl Zeiss, Oberkochen, Germany) and a Nikon 1 J5 camera. The feed solution was circulated from the feed container to the filtration module using an OSMO inspector system (CONVERGENCE, NL) which records pressure and flow data. A schematic drawing of the system with microscope and camera is presented in Figure 1. 

**Figure 2:**
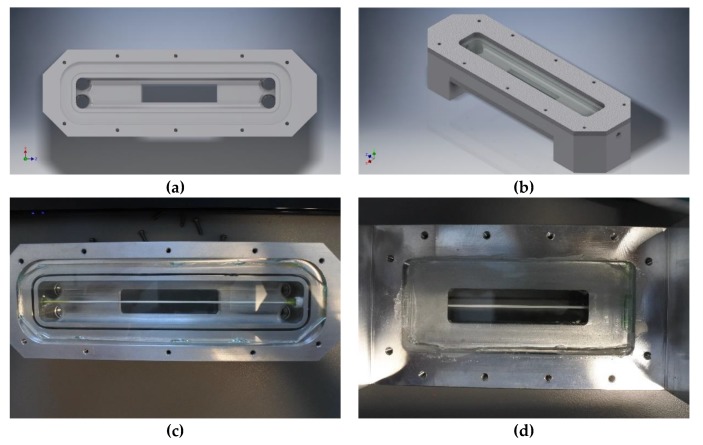
Three dimensional renderings of filtration cell without (**a**) and with (**b**) glass and mounting plates and pictures of disassembled (**c**) and assembled filtration module (**d**) with membrane.

The crossflow module is depicted in Figure 2 and was made of a custom CNC’ed aluminum block with a flow volume of 160 × 15 × 5 mm. In the top and bottom of the membrane module a 3 mm glass pane was fastened to allow the microscope to see through the assembly. A single 150 mm long piece of hollow membrane fiber (SFX 2860xp 30 nm with polyvinylidene fluoride active layer) was placed lengthwise in the module. To ensure the best observation conditions, the membrane was pulled taught to minimize movement. The membrane was sealed on one end allowing permeate withdrawal from one side. The videos were taken using a 10× objective lens at a resolution of 400 × 144 px at 1200 fps and the scale of the video was calibrated using a micro ruler. 

### 2.2. Filtrations

Monodisperse core-shell particles with a polystyrene core and with a hairy shell of polyacrylic acid (negatively charged) or hydroxypropyl cellulose (neutral) was synthesized with the method described in Lorenzen et al. [17]. The particles were used as model foulants and had an average diameter of 1.6 μm, a density of 1050 kg/m^3^ and a zeta potential of −1.31 mV (low/no charge) and −40.47 mV (high negative charge). The solution was made daily by adding 0.03 g particles to 5 L of tap water. The solution was stirred and then circulated 15 min in the filtration system by pumping prior to the filtration analysis.

Filtrations were carried out on suspensions of both low charge and high charge particles. Each suspension type was filtered at room temperature (21 °C), 1 bar and 2 bar TMP and crossflows of 60 kg/h and 160 kg/h, corresponding to average crossflow velocities of 0.13 and 0.33 m/s in the membrane module, respectively. For each experiment, three repetitions were conducted to find the reproducibility of the results. The permeate was collected in a beaker on a balance (Kern PCB 6000-1, Kern & Sohn GmbH, Balingen, Germany) for on-line data collection. From the measured permeate mass, the permeate flow and flux was calculated.

### 2.3. Microscopy and Video Analysis

The recorded video was converted in VLC Media Player to MP4.h265 format with an added sharpness and graduation filter to make it easier to find the particles using MATLAB. A detailed description of the particle track and trace procedure is presented in Appendix A. The particles were identified using an area comparison function in MATLAB, and the center points were then saved. A geometric overlay function was used on each particle in each frame to test if there was a particle close by in the next frame. If they were within the specified geometry it was considered a trace. A trace can be multiple frames long. If multiple particles were in the geometric target area the last one found were saved. Traced data was filtered to allow for data analysis, by (1) removing traced articles that were detected in less than 5 frames, (2) removing the last registered position of a traced particle, and (3) by removing traces that did not move more than 20 μm in the length direction (in order to remove erroneously tracked membrane).

Particle positions put into length coordinates along the membrane (*x* coordinate) and height coordinates from the membrane (*y* coordinate), where lengths and heights has been calculated from pixel positions, using a 1.26 μm distance between particles.

The velocity of particles moving along the fiber, *v*_x_, and velocity of particles moving towards the fiber, *v*_y_, was calculated using the following equations.

*v*_x,I_ = (*x*_i+1_ − *x*_i_)/Δ*t* (1)

*v*_y,i_ = (*y*_i_ − *y*_i+1_)/Δ*t* (2)

where *v*_y,i_ and *v*_x,i_ are the velocities along and towards the membrane (longitudinal and perpendicular velocities, respectively) for the particle tracked in frame *i*. *x*_i_, *x*_i+1_, *y*_i_ and *y*_i+1_, are the positions of tracked particles in frame *i* and *i+1* and Δ*t* = 1/1196 fps = 0.84 μs is the time difference between each frame. Accordingly, the acceleration of particles towards the membrane, *a*_y_, was calculated using the following equation:

*a*_y,i_ = ((*y*_i−1_ − *y*_i_)-(*y*_i_ − *y*_i+1_))/Δ*t^2^* (3)

### 2.4. Computational Fluid Dynamics Simulation

A three dimensional method to model the flow in the filtration cell was set up using the commercial software Comsol Multiphysics 5.4. The 3D renderings shown in Figure 2a,b were used for simulations. The water flow velocity through the cell was simulated at *T* = 20 °C and 160 kg/h flow of water through the cell. The boundary condition for the membrane was set as a fixed flow outlet with the flow set at the measured permeate flow of 0.037 mL/s. The outlet was set as a pressure outlet at 2 bar and the inlet was the inlet velocity which was 0.33 m/s. The mathematical model used was the *κ-ε* shear stress transport (SST) model using Low Reynolds wall treatment. To make the model solvable with the flow resolution needed the mesh was split into 3 parts. At the observable area, a 1 × 1 × 1 mm cube was placed with an extremely fine mesh to get the best possible wall resolution. The membrane fiber had a less fine mesh and the bulk flow and other walls had an optimized mesh. This was necessary because of the limited hardware available. Multiple different passes were done with different meshes to ensure that the meshing was adequate to resolve the flow. The membrane surface was set to smooth. 

## 3. Results

### 3.1. Flow Simulations

**Figure 3:**
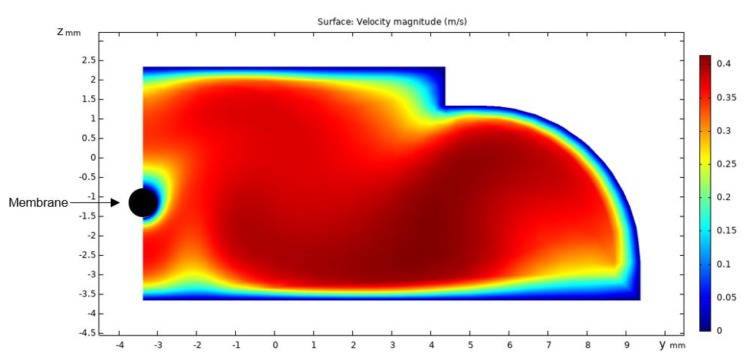
Surface velocities in the right half cross sectional plane of the filtration cell calculated by CFD modelling using a crossflow of 160 kg/h and 2 bar TMP.

The water velocity profile of the one half of the flow cell obtained from CFD simulation at 0.33 m/s mean crossflow velocity is shown in Figure 3.

The figure shows that the velocity approaches 0.40 m/s in the cell and decreases towards the walls of the cell and the membrane. The velocity increases with the distance from the membrane and reaches 0.35 m/s at a distance of 1 mm from the membrane surface.

### 3.2. Particle Track and Trace

**Figure 4:**
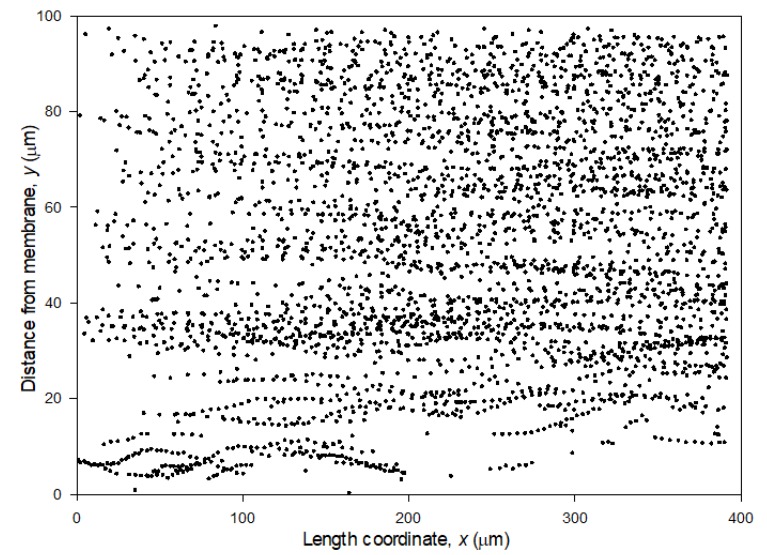
Tracked particles during filtration at different length coordinates along the membrane (*x* coordinate) and height coordinate from the membrane (*y* coordinate). The data in the figure is from filtration of lowly charged particles at 2 bar and crossflow velocity of 0.13 m/s and collected over 3 s filtration.

Core-shell particles were filtered and the filtration process monitored with DO. The videos were analyzed in MATLAB. Figure 4 shows a representative map of particles tracked near a membrane during filtration of lowly charged particles at TMP = 2 bar and crossflow velocity 0.13 m/s. The plot shows a collection of all the identified particles during 3 s filtration. The same particle can be recorded several times. The position is given in distance from the membrane, *y*, vs the length coordinate along the membrane, *x*. The particles moved with the crossflow from left to right i.e., from low to high *x*.

**Figure 5:**
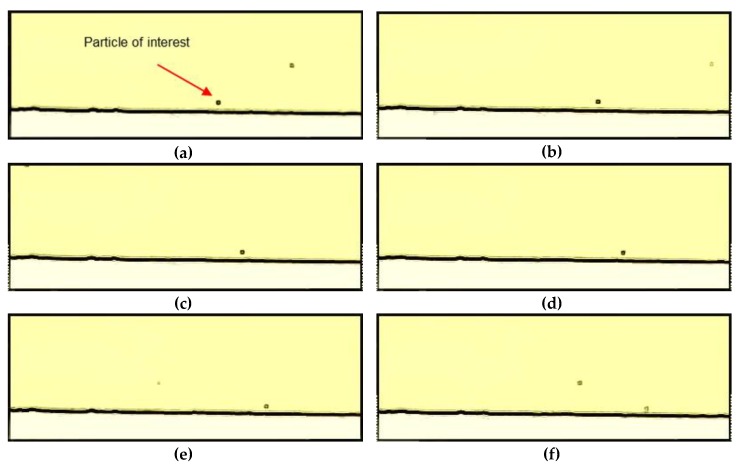

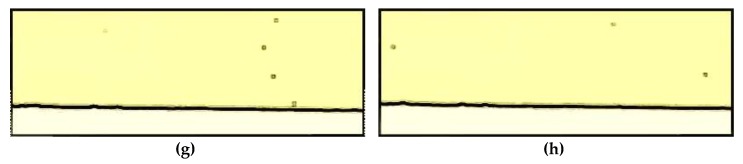
Graphs (**a**)–(**h**) show frames in chronological order. The frames show how particles moves from left to right along the membrane (in the bottom of each frame) during filtration of lowly charged particles at a crossflow velocity of 0.33 m/s and 2 bar TMP. The difference in time between the frames is 0.2 s.

As observed from Figure 4 there seem to be less particles near the membrane (less than 20 μm) than far from the membrane (more than 40 μm from the membrane). Close to the membrane, the particles can easily be identified and tracked as they move along the membrane, and it is observed how they oscillate in distance from the membrane, *y*. At distances higher than 20 μm from the membrane there is a more chaotic map of particles. In the video uploaded under supplementary material it can be observed how particles approach the membrane. Once they reach a critical distance of approximately < 5 μm, from the membrane, the particle velocity decline and the particle falls into the membrane and deposits. This is illustrated in Figure 5 showing frames captured from the video with 0.2 s intervals. 

**Figure 6:**
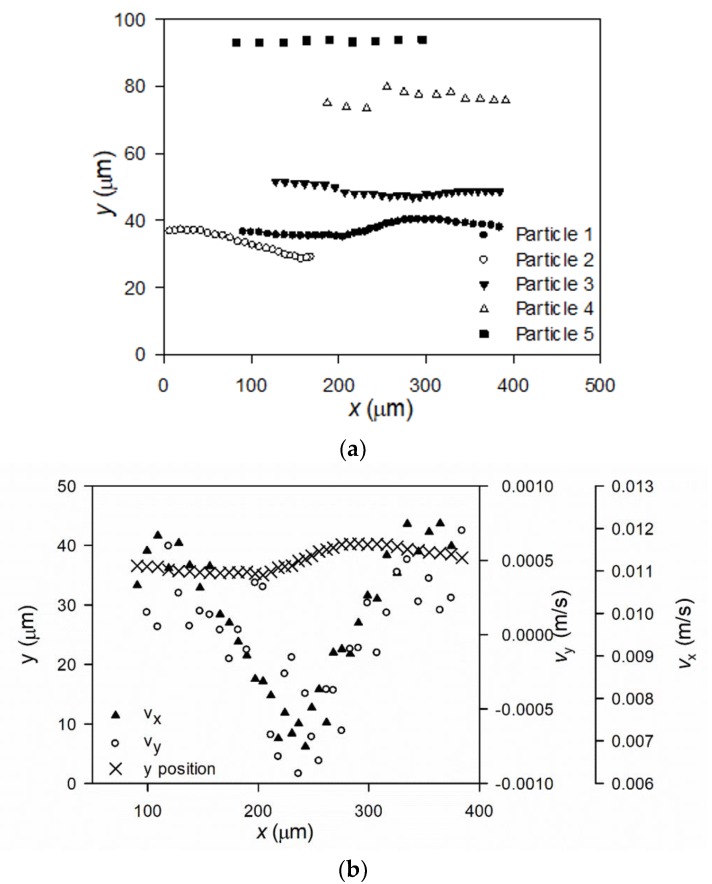
Particle movements of five tracked particles from recordings of filtrations conducted with lowly charged particles filtered at 2 bar and a crossflow velocity of 0.13 m/s (**a**). Plot (**b**) shows the movement of Particle 1 from (**a**) along with the calculated velocity of the particle along and towards the membrane fiber.

Figure 6a shows how five different particles selected from Figure 4 are tracked and move along the membrane with the crossflow.

The graph shows how the five different particles’ positions are tracked and mapped as they move along the fiber. Particles seem to appear and disappear, e.g., Particle 2 is no longer observed at x > 180 μm, while Particle 4 appears at 190 μm. This is explained by particles moving in the third dimension, z, towards or away from the camera, hence it comes out of focus to be captured. The MATLAB script filtrates data to show only particles that have been tracked for at least five frames. 

As observed from Figure 6a, the particles far from the membrane surface (e.g., Particle 4 and 5) move across the membrane with the same distance from the membrane. Particles moving closer to the membrane alternate from advancing towards and retreating from the membrane surface. During the 3 s video recordings, the particles near the membrane are detected a higher number of times than particles far from the membrane. Also, the distance between particle positions between each frame seems lower for particles near the membrane surface, showing that the velocity of particles along the membrane is slower, the closer they are to the membrane. 

In Figure 6b, the position of Particle 1 (from Figure 6a), velocity along the membrane, *v*_x_, and towards the membrane, *v*_y_, is plotted as function of the length coordinate along the membrane. This shows that the particle longitudinal velocity decreases as the particle advances towards the membrane. As the particle retreats, the longitudinal velocity increases again. Hence, the tracking methodology developed with high speed DO shows a clear influence of particle position on the longitudinal particle velocity. The particle’s velocity in the *y*-dimension changes from being positive, i.e., the particle advances towards the membrane, to being negative, i.e., the particle retreats from the membrane, and then again moves away from the membrane. It is observed that *v*_y_ << *v*_x_, which is a consequence of the high crossflow velocity (0.13 m/s in the experiment behind Figure 5) compared to the permeate flux, which was measured to be 351.8 ± 13.7 LMH, i.e., 9.8 · 10^−5^ ± 0.38 · 10^−5^ m/s, during filtration at 2 bar TMP. At 1 bar TMP filtrations, the permeate flux was measured to 263.0 ± 1.9LMH, i.e., 7.3 · 10^−5^ ± 0.05 · 10^−5^ m/s. The permeate flux was not affected by crossflow velocity, as almost no fouling was formed with the low concentration of particles.

### 3.3. Longitudinal Velocity Profiles

**Figure 7:**
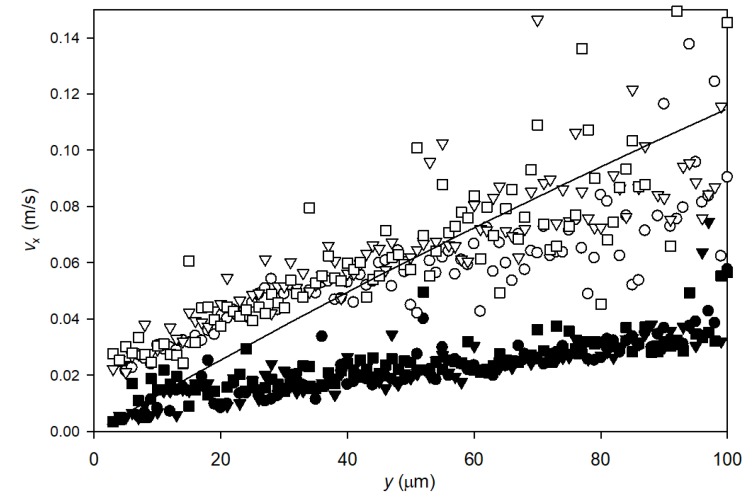
Longitudinal velocity of lowly charged model particles along the membrane surface plotted against particle distance from the membrane surface. Points ■, ● and ▼ represent particles recorded at a crossflow velocity of 0.13 m/s while □, ○ and ∇ are particles recorded at 0.33 m/s crossflow velocity. For each crossflow velocity, three recordings were conducted during membrane filtration at TMP = 2 bar. The line represents modelled water velocities along the membrane fiber at 0.33 m/s crossflow velocity and 2 bar TMP using COMSOL.

The longitudinal velocity of particles along the membrane was calculated using Equation (1) for each particle tracked in the video from filtration experiments. The velocities were averaged within 1 μm intervals in distance from the membrane, *y*, and plotted against *y*. The velocity profiles of lowly charged particles during filtrations at 2 bar TMP and crossflow velocities of 0.13 m/s (three filtrations) and 0.33 m/s (three filtrations) are plotted in Figure 7.

The longitudinal velocity of particles increases with higher distance from the membrane surface and the velocity of particles tracked during the 0.13 m/s crossflow experiments is lower than the velocity of particles tracked during the 0.33 m/s crossflow experiment (Figure 7). The results are reproducible, as the three experimental runs of each setting showed similar velocity profiles. 

CFD simulations were used to calculate the profile of fluid velocity as function of distance from the membrane during filtration at 2bar TMP and crossflow 0.33 m/s. There was good correlation with theoretically modelled fluid flow and with measured particle velocity with some underprediction of flow close to the membrane and overprediction far from the membrane. The longitudinal velocity profiles obtained at a crossflow velocity of 0.33 m/s did not reach the expected bulk particle flow velocity within a distance of 100 μm from the membrane, as also predicted from the CFD simulation.

Close to the membrane there is no data for measured longitudinal velocity for particles (<3 μm). This is in accordance with the observation from Video S1 in Supplementary Material that when particles move close to the membrane, they reach a position where they deposit instead of being transported with the longitudinal crossflow. This may be observed as a stagnant layer in which crossflow does not affect particle movement. The extent of the concentration polarization layer can also be determined theoretically as described in Appendix B using the approach described in Christensen et al. [18]. Using the measured flux at 2 bar TMP, 352 LMH, a particle diameter of 1.6 μm, and a crossflow velocity of 0.33 m/s, the theoretical thickness is determined to δ = 0.13 μm, which is well below the observed thickness of the stagnant layer.

**Figure 8:**
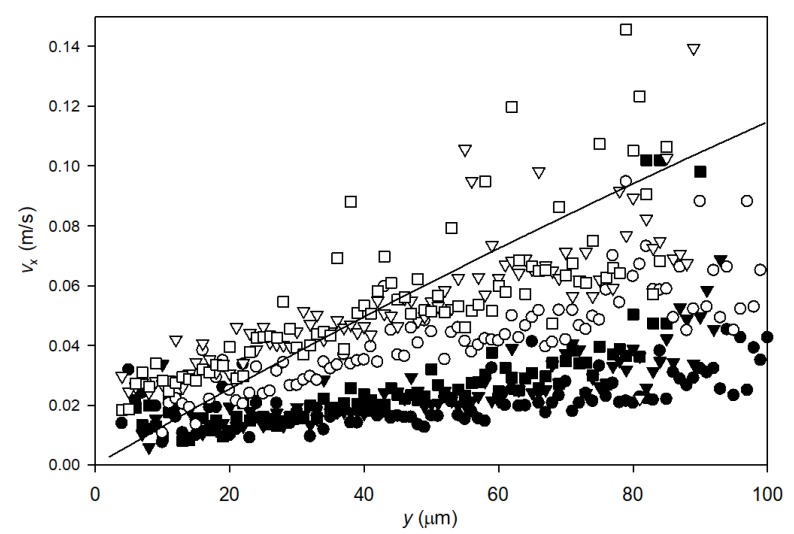
Speed of highly charged particles along the membrane surface plotted against particle distance from the membrane surface. The points ■, ● and ▼ represent velocities of particles recorded at a crossflow velocity of 0.13 m/s while □, ○ and ∇ are obtained from particles recorded at 0.33 m/s crossflow velocity. For each crossflow velocity, three recordings were conducted during membrane filtration at TMP = 2 bar. The line represent modelled water velocity along the membrane fiber at 0.33 m/s crossflow velocity and 2 bar TMP using COMSOL.

Figure 8 shows the longitudinal velocity profiles for tracked particles during filtration of high charge particles at TMP = 2 bar and crossflow velocities of 0.13 and 0.33 m/s. These show the similar trends as for the low charge particles. Hence, particle interactions (repulsion and attraction) do not influence flow profiles along the membrane in the 4–100 μm distance range analyzed in this study. This is well in accordance with DLVO theory, as the Debye length can be estimated to be only 2.6 nm by assuming an ionic strength of the tap water of 0.013 mol/L. The assumed ionic strength was based on water analyses by the local water utility company.

Velocity profiles of particles tracked under 1 bar TMP filtrations (data not shown) showed the same tendencies as for 2 bar TMP filtrations, hence neither TMP nor particle surface charge affected particle’s longitudinal flow profiles. 

### 3.4. Perpendicular Velocity Profiles

**Figure 9:**
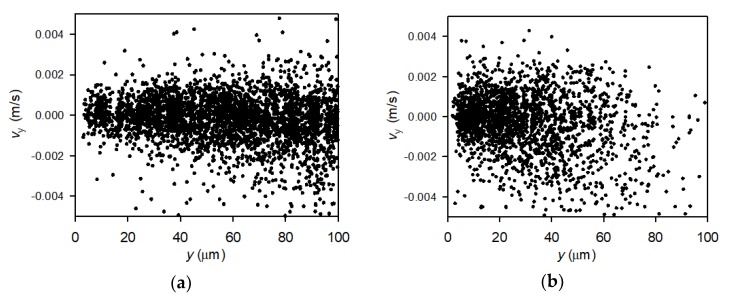
Velocities of particles towards and away from the membrane recorded during filtration of particles of low negative charge at 2 bar TMP and crossflow velocity of 0.13 m/s (**a**) and 0.33 m/s (**b**).

The perpendicular velocities, i.e., the speed of particles directly towards or away from the membrane, was calculated using Equation (2). Figure 9a,b show the particle velocities towards the membrane at varying particle positions near the membrane during filtration of lowly charged particles at TMP = 2bar and 0.13 m/s and 0.33 m/s crossflow velocity, respectively. 

Figure 9a show data for an experiment with a crossflow velocity of 0.13 m/s. The measured velocities towards the membrane (−0.004 m/s–0.004 m/s) are orders of magnitude lower than the measured velocities of the particles along the membrane (up to 0.1 m/s), which prevents rapid deposition. Secondly, it is observed that the velocity of particles moving towards the membrane from the permeation drag is counteracted by particles moving out from the membrane towards bulk suspension. The velocity of particles moving towards the membrane is measured to maximum 0.004 m/s, which corresponds to a permeate flux of up to 14400 L/m^2^/h (LMH). This is higher than the actual permeate flux measured at TMP = 2 bar, which were only 353 ± 14 LMH. Although particles move with a high velocity towards the membrane, a low amount of deposition on the membrane is observed in the videos, which is in accordance with the stable flux throughout filtrations. If particles deposited on the membrane, a lower drag of particles towards the membrane would be observed due to reduced permeability. The lower-than-expected observed velocities of particles moving towards the membrane suggests that (1) the hydrodynamic around the membrane disturbs the fluid flow eventually due to vibration of the membrane or (2) the forces acting on the particles change during the particle transportation, e.g., due to particle rotation. The surface of the membrane was monitored during the experiment and no vibration was observed, still the fluid flow may be disturbed around the filter and cause small fluctuation of the particle velocity. Also, forces acting on the particle may change. If this is correct, the permeation drag used in literature to quantify foulant transport to a membrane surface significantly underestimates the real rate of transport and the forces may vary more than expected. Another explanation might be that the permeation drag can only describes particle transport rate towards the membrane close to the membrane, e.g., a distance < 5–10 μm which is where the particle starts deposition as described in Section 3.2. 

Figure 9b shows data obtained at filtration with a crossflow velocity of 0.33 m/s. There is only particles moving towards and away from the membrane within a distance of up to 80 μm (critical distance) from the membrane surface. At this critical distance, the velocity of particles along the membrane is approximately 0.075 m/s (Figure 7). Comparing with the perpendicular velocities obtained at 0.13 m/s crossflow filtrations in Figure 9a shows that the apparent critical distance has not been reached, which may be a consequence of the crossflow not reaching a critical level to eliminate transport towards and away from the membrane within the first 100 µm from the membrane (Figure 7). It was expected that a higher crossflow velocity, which was reached at longer distances from the membrane, would counteract the relatively low transport of particles towards the membrane, hence that perpendicular transport would decrease with distance from the membrane, but the abrupt absence of perpendicular transport at distances > 80 μm was not expected. Alternatively, the lack of transport towards the membrane of particles further from the membrane than 80 μm could also be a result of an inadequate amount of frames of tracked particle a high distances from the membrane and at high crossflow velocity, e.g., there is not enough captured particles to determine a perpendicular velocity.

The measured perpendicular velocity profiles suggest that particle movement is more complex and chaotic and underestimates local velocities of particles moving towards and away from the membrane. With the dimensions and flows along the membrane used in this study, the results can be transferred to e.g., flat sheet and spiral wound microfiltration, whereas the flow around close packed fibers in hollow fiber modules may differ from the flows investigated in this study. This puts demand for further understanding of the mechanisms of particle movement in membrane filtration at lab scale (idealized conditions) and full scale (real conditions), which should be described in detail in further studies.

**Figure 10:**
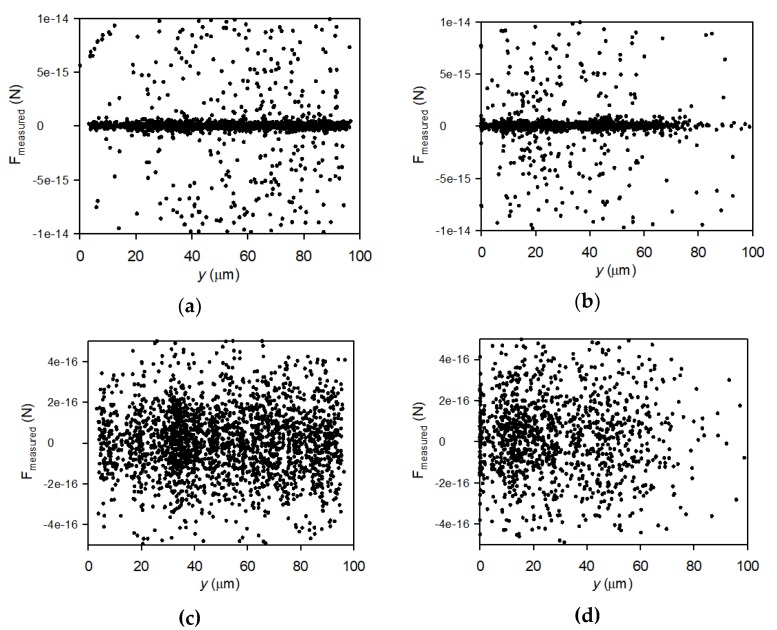
Measured kinetic forces of lowly charged particles towards the membrane plotted vs distance from the membrane surface during filtration at 2 bar TMP and crossflow velocity of 0.13 m/s (**a,c**) and 0.33 m/s (**b,d**).

The measured force of particles moving towards and away from the membrane can be determined by first finding the acceleration of each particle moving towards and away from the membrane by use of Equation (3). The mass of particles was estimated from the volume of a sphere with diameter of 1.6 μm and density 1050 kg/m^3^ relative to the density of water (998 kg/m^3^). Accordingly, the force of particle movement can be found as the product of the particle mass and acceleration and plotted against distance from membrane in Figure 10. 

The measured particle forces are in the range −1 × 10^−14^ N to 1 × 10^−14^ N, positive if they accelerate towards the membrane and negative if the accelerate away from the membrane. However, Figure 10c,d shows that most particle forces are in the range −4 × 10^−16^ N to 4 × 10^−16^ N. The trend in forces is the same as for velocities, as there for the 0.33 m/s filtrations also is a critical distance at around 80 μm above which there are no forces acting on the particles towards or away from the membrane.

The theoretical lift force, *F*_L_, transporting particles away from the membrane can be estimated by the following Equation (4):

*F*_L_= 0.761τ_w_^1.5^ · *a*^3^ · *ρ*^0.5^/η (4)

in which *a* is the particle diameter (1.6 μm), *ρ* is the density of water (998 kg/m^2^), and *η* is the dynamic viscosity of water (1.002 × 10^−3^ Pa · s). The wall shear stress (*τ*_w_) was determined to *τ*_w_ = 1.28 Pa by multiplying the gradient in fluid velocity with distance from the membrane (*du/dy* = 1280 1/s within 0–10 μm) at a crossflow of 0.33 m/s (determined from CFD simulations). The resulting lift force is 1.43 × 10^−13^ N, i.e., a factor 10 larger than the measured forces of which particles move away from the membrane (Figure 10b,d). Thus, lifting forces may also play a role in the particle velocity and change of particle velocity, but are counteracted by the gravity force, i.e., the drag of particles by permeation. 

### 3.5. Particle Count Profiles

**Figure 11:**
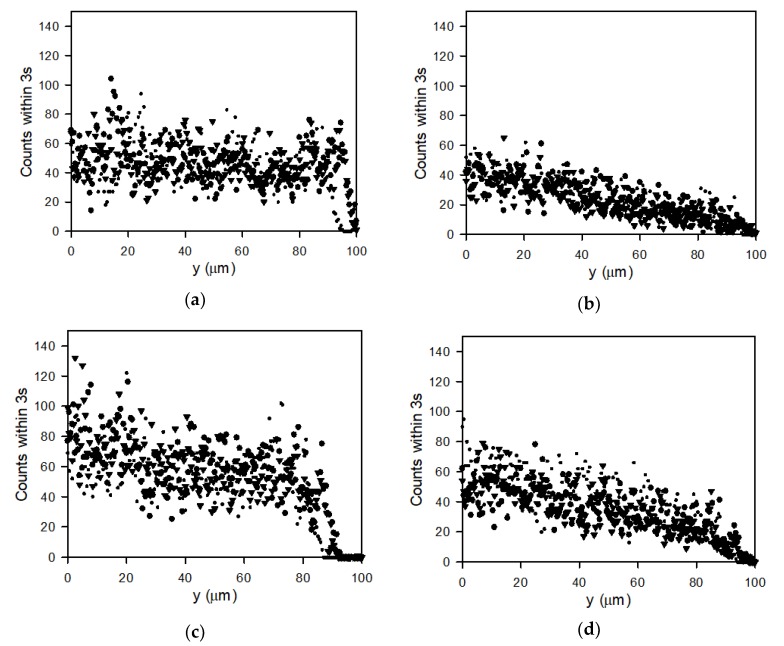
Graphs showing number of particle counts during 3 s filtration videos plotted against distance from the membrane surface. Data are obtained from recorded filtrations at TMP = 2 bar for (**a**) lowly charged particles at 0.13 m/s crossflow velocity, (**b**) highly charged particles at 0.13 m/s, (**c**) lowly charged particles at 0.33 m/s crossflow velocity and (**d**) highly charged particles at 0.33 m/s crossflow velocity. Each plot contains three different plots for the three filtrations performed at each setting (■, ● and ▼).

The number of particles during 3 s of filtration was counted and plotted against distance from the membrane in Figure 11 for lowly (a,b) and highly (c,d) charged particles filtrated at 2 bar TMP and crossflow velocities of 0.13 m/s (a,c) and 0.33 m/s (b,d). It should be noted that particles far from the membrane will be replaced faster than particles close to the membrane. 

Comparing the plots in Figure 11a,b shows a steady distribution of lowly charged particles from the membrane for the lower crossflow velocity, while for the higher crossflow velocity, the number of particles declines towards reach zero near *y* = 100 μm. For the higher charge particles there seems to be a higher total count of particles (Figure 11c), suggesting a higher concentration of particles. However, there is a decline in count with distance from the surface even at the low crossflow velocity, which may be due to repulsion between the charged particles. 

## 4. Conclusions

A procedure was developed to record and analyze particle distribution and movements along and towards a membrane during crossflow microfiltration. The particles were tracked and traced using video recordings through a microscope with a high speed camera. Particle velocity along the membrane was measured and showed good correlation with fluid velocity along the membrane determined by CFD simulations and showed higher velocities for experiments conducted at higher crossflow velocities. The particle velocity was independent of TMP and particle surface charge. 

The velocity of particles towards and away from the membrane was significantly higher than the measured permeate flux, which in literature is frequently used to quantify foulants transport towards the membrane. This was only observed within a region close to the membrane. The extent of this region decreases with higher crossflow velocity. The high velocity towards the membrane is counteracted by similar particle velocities away from the membrane. This may be due to distribution of the fluid flow close to the fiber. Close to the membrane, the particle velocities shift direction, which is not described in classical filtration theory. An explanation may be that the shift in velocity is an effect of particle rotation, membrane vibration or shift in flow pattern close to the membrane. Furthermore, the lifting force may also have an influence but was significantly higher than the particle force estimated from the acceleration of the particles. Hence, the results suggest that the mechanism of particle deposition is more complex than described in literature today, as the particles move in a highly chaotic manner, even at low crossflow velocities with local velocities towards the membrane exceeding that predicted by the permeation drag. This calls for a revised understanding of particle behavior during crossflow filtration. 

Finally, particle counting enabled the determination of particle distribution profiles away from the membrane surface, which confirms and quantifies higher particle accumulation near the membrane for lower crossflow velocities. The determination of particle distribution and velocity profiles with the procedure developed in this study is a promising tool to study the behavior of particles near the membrane before they deposit and form fouling layers. 

## Figures and Tables

**Figure A1:**
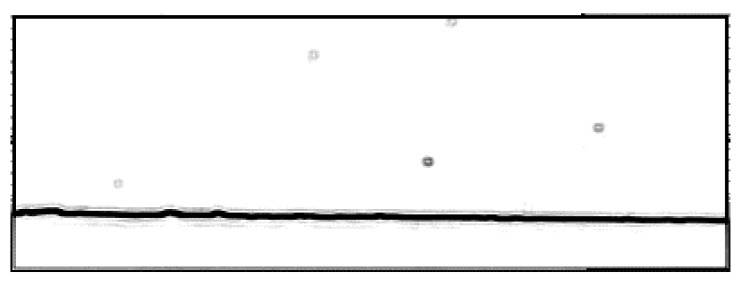
Representative filtered frame with visible particles.

## Supplementary Materials

The following are available online at https://www.mdpi.com/2077-0375/10/4/68/s1.

## Appendix A

### MATLAB Particle Tracking

To analyze what is considered a particle in MATLAB, the videos were first converted with a gradient and sharpness filter in VLC Video Player. Figure A1 shows an example of a post filter frame.

To track the particles in MATLAB the individual frames were converted to grayscale using the “imbinarize” function and anything below a set threshold were set to black. Then using “imdilate” anything that is not background is dilated so all particles are a solid color. “imopen” is then used to draw circles around the particles making them more regularly shaped. This is done in the following lines of code:

img = img(:, :, 3);
img = ~imbinarize(img, ‘adaptive’,’Sensitivity’,0.7);
img = imdilate(img, strel(‘disk’, 1));
img = imopen(img, strel(‘disk’, 1));

Afterwards the center of the particles is found and it is determined what is a particle and what is noise. The function “regionprops” is used to find the center of all circles and measure their area. All circles above a certain minimum area then have their center coordinate saved as a result. This is done in the following code:

p = regionprops(img, {‘Centroid’, ‘Area’});
ind = [p.Area] > minsize;
cent = reshape([p.Centroid], 2, length(ind));
cent = cent(:, ind);

This code is repeated for every single frame in a single video. To help find the correct minimum area a plot of the frame and what is shown for testing purposes however that is too slow for actually doing a whole video and is commented out normally. Additionally, rotation and cropping was added in the code to align the membrane horizontally and to minimize runtime.

### MATLAB Particle Tracing

To trace the particles and find them in all frames of the video the center points given from the particle analyzing script was used. Around any given particle center point a polar coordinate system is made and a function is drawn around it. Through testing the following functions were used:

L = linspace(0,2*pi,6); –sets up graph space
xv = ParPos(frame).xy(1,particlenumber) – 30 + cos(L)*50’; -x part of polar function
yv = ParPos(frame).xy(2,particlenumber) + 2 + sin(L)*5’; -y part of polar function

Notice in the “linspace” function it is only drawing 6 points, effectively drawing a hexagon. Increasing this number gets it closer and closer to a circle. However, it also increases computation time. The function for x (−30 + cos(L)*50) and y (+2 + sin(L)*5) was chosen to draw the polygon that had the best chance to include only the correct particle in the following frame.

The “inpolygon” function is used on the just made polygon to test if any particle centers from the next frame is inside the polygon. If there are, they are saved. This is done in the following code:

Xq = ParPos(frame+1).xy(1,:); –x position for all particles in the next frame
yq = ParPos(frame+1).xy(2,:); –y position for all particles in the next frame
[in] = inpolygon(xq,yq,xv,yv); –Here the particles are tested if they are inside the polygon

The xq and yq is the particle center positions for all particles in the following frame. The results of the test are either a true or false for all particles tested and are saved in the vector “in”.

If any particles are found inside the polygon they need to be saved as a trace. That is done in the following “for” loop:

for lengthin=1:length(in) –Makes a loop that goes through the length of “in”
lengthins=lengthin;
if in(lengthin)==true –Goes through the “in vector and checks for points in the polygon
if ismember(ParPos(frame).xy(1,particlenumber),tracer(:,:))==true -Tests if original particle has been seen before
[row,col]=find(tracer==ParPos(frame).xy(1,particlenumber));
kolonne=col;
tracer(row(end),col(end)+1)=ParPos(frame+1).xy(1,lengthins);
tracer(row(end)+1,col(end)+1)=ParPos(frame+1).xy(2,lengthins);
else -If they haven’t been found before both particles are saved here
tracer(tracenumber*2-1,1)=ParPos(frame).xy(1,particlenumber);
tracer(tracenumber*2,1)=ParPos(frame).xy(2,particlenumber);
tracer(tracenumber*2-1,2)=ParPos(frame+1).xy(1,lengthins);
tracer(tracenumber*2,2)=ParPos(frame+1).xy(2,lengthins);
tracenumber=tracenumber+1;
end
end
end

The “for” goes through the “in” vector made in the “inpolygon” and the “if” functions finds if any particle were in the polygon. If there is a particle in the polygon the “ismember” function test to find if the testing particle is part of a previous trace, if it is the new particle position is saved there. If it has not been saved before, a new trace is made containing both the testing particle and the new particle. If there are multiple particles inside the polygon the last particle found inside the polygon is saved as it simply overwrites the previous found particles. No evaluation of what is the “best” fit particle is made and would be a good addition should further work be made. This could easily be added as a test before the “for” loop.

The testing polygon shape was decided through testing and for that a figure was made to show the polygon and particles in the next frame. However, that was commented out when running the script normally to make it possible to actually complete the script.

### MATLAB Scripts and Their Functions

The following MATLAB scripts are used to analyze particle flow and distributions.

- videotracking.m: Tracks particles in converted videos. Input: Filtered video file directory. Output: Particle positions “ParPos”.
- tracing.m: Traces particle positions. Input: particle positions “ParPos” from videotracking.m. Output: Traced particle data “tracer”.
- Filterfunction: Filters tracked particles. Input: “tracer”. Output: “tracer”.
- Velocities.m: Calculates velocities along and towards the membrane. Input: “tracer”. Output: “Velocities”.
- PlotParticles.m: Plots traced particles and saves their position in Excel file.. Input: “tracer”. Output: “ALLPAR”, Excel file and Plot of particle positions.
- Plots.m: Generates plots of particle velocities and saves velocity data. Input: “tracer”. Output: Plots and Excel file.

## Appendix B

The thickness of the concentration polarization layer is estimated using the following equation:

*δ* = *D/J* · ln(C.P.) (A1)

where *D* is the diffusion coefficient (sum of shear induced, *D*_S_, and Brownian diffusion, *D*_B_, coefficients. C.P. is the concentration polarization modulus, which is assumed to be 20.

*D*_S_ and *D*_B_ are determined from:

*D_S_* = 0.5(ϕ · a/2)^2^ · *τ*_w_/μ (A2)

*D_B_* = k_B_ · T/(3π · μ · a) (A3)

where ϕ is the solid volume fraction (assumed to be 0.01), *τ*_w_ = 1.28 Pa, *k_B_* is the Boltzmann constant and *T* is the temperature (294 K).
